# Lipid asymmetry and membrane trafficking: Transbilayer distribution of structural phospholipids as regulators of exocytosis and endocytosis

**DOI:** 10.1016/j.jbc.2025.110441

**Published:** 2025-07-02

**Authors:** Margherita Caputo, Olga Gubar, Petra Tóth, Nicolas Vitale, Stéphane Gasman, Stéphane Ory

**Affiliations:** 1 Centre National de La Recherche Scientifique, Université de Strasbourg, Institut des Neurosciences Cellulaires et Intégratives, Strasbourg, France; 2 IEO, European Institute of Oncology IRCCS, Milan, Italy

**Keywords:** exocytosis, endocytosis, cholesterol, flippase, floppase, membrane asymmetry, phospholipids, scramblase, PLSCR1

## Abstract

The plasma membrane of eukaryotic cells is highly dynamic and asymmetrically organized. Its continuous remodeling plays a crucial role in diverse cellular processes, including apoptosis, blood coagulation, and vesicular trafficking. The distribution and rearrangement of phospholipids (PLs) within the bilayer are tightly regulated, influencing membrane curvature, tension, and organization. This review examines the role of PL asymmetry in vesicle fusion, the final step of exocytosis, and in vesicular membrane retrieval by compensatory endocytosis in neurosecretory cells, with a particular emphasis on structural PLs such as phosphatidylcholine (PC), phosphatidylethanolamine (PE), and phosphatidylserine (PS). We discuss the molecular mechanisms that maintain and disrupt PLs asymmetry and explore how lipid rearrangements affect vesicle dynamics. Additionally, we highlight recent findings on lipid scramblases, particularly phospholipid scramblase-1 (PLSCR1), and their role in regulated exocytosis and compensatory endocytosis.

The membranes of living organisms are composed of a complex mixture of lipids that self-assemble into bilayers and embed proteins to form organelles and cell boundaries. The bulk of the membrane lipids consists of phospholipids (PLs), which are categorized into two groups: glycerophospholipids with a glycerol backbone and sphingophospholipids with a sphingosine backbone. Phosphatidylcholine (PC) and phosphatidylethanolamine (PE) constitute the major classes of glycerophospholipids, whereas phosphatidylserine (PS), phosphatidylinositol derivatives (PIs), and phosphatidic acid (PA) are present in lower proportions. Glycosphingolipids (GSLs) form a diverse family of sugar containing sphingolipids, whereas sphingomyelin (SM), is the predominant sphingophospholipid of the plasma membrane. Among these, the most abundant PLs are considered structural, defining the membrane topology. Whereas less abundant, typically, the anionic PLs act as signaling molecules through their ability to interact with specific proteins.

Despite their amphipathic nature and low spontaneous translocation rate (1), PLs distribution is asymmetric in most bilayer membranes of eukaryotic cells. The concept of lipid asymmetry was first introduced by Bretscher in 1972 (2). In this model, SM, GSLs and PC predominantly reside in the outer leaflet, whereas aminophospholipids such as PS and PE are mainly found in the inner leaflet. Subsequent studies confirmed that the PL distribution observed in erythrocyte plasma membrane (PM) is broadly applicable to all animal cells (3, 4). Advanced mass spectrometry has since confirmed this asymmetric lipid organization of human erythrocyte membranes with unprecedented detail of its composition by selectively digesting exoplasmic leaflet lipids and inferring the inner leaflet composition (5).

The plasma membrane is continuously reorganized to adapt to cellular status as well as to environmental cues and mechanical constraints. For instance, these dynamic processes occur at the plasma membrane during exocytosis and endocytosis, which are crucial for maintaining cellular homeostasis and enabling adaptation to external stimuli. Exocytosis is the process by which vesicles fuse with the plasma membrane to release their contents. Two types of exocytosis coexist: (i) constitutive exocytosis, which involves the continuous delivery of lipids and proteins to the plasma membrane and does not require external signals to initiate vesicle fusion, and (ii) calcium-regulated exocytosis, which is restricted to specialized cells that timely release hormones or neurotransmitters in response to an intracellular calcium rise. The core mechanisms by which vesicles deliver or release secretory products are highly conserved and involve sequential steps. After budding from the trans-Golgi and their transport to the cell periphery, vesicles undergo tethering, docking, and ultimately fuse with the plasma membrane. While these mechanisms involve successive reorganization of PLs across different membrane compartments, most studies have focused on the role of protein complexes in membrane fusion, with comparatively less attention paid to lipid rearrangements and their functional consequences.

Exocytosis implies the supply of vesicular membrane to the plasma membrane, perturbing plasma membrane homeostasis through an increase in surface area and changes in mechanical properties. In most biological processes requiring extensive exocytosis, such as cell migration, neuronal growth cone extension, phagocytosis or tip growth in the fission yeast, the supply of membrane is coupled with endocytosis to generate a bulk membrane flow necessary for the redistribution of proteins and lipids, facilitating the associated morphogenetic events (6, 7). This balance between exocytosis and endocytosis is best demonstrated in migrating cells, where the overall cell surface remains constant due to predominant endocytosis at the trailing edge and extensive exocytosis at the leading edge. Current models suggest that this spatial segregation of membrane trafficking is regulated by fluctuations in membrane tension that rapidly propagate within the cell (8). During neurosecretion, compensatory endocytosis fully compensates for exocytosis, effectively replenishing vesicular pools and selectively retrieving vesicular components from the plasma membrane, a mechanism particularly critical during sustained neurotransmitter release induced by repetitive stimulation (9, 10). Exocytosis and endocytosis are tightly coupled in space and time, and as in cell migration, membrane tension has recently emerged as an important regulatory mechanism (11, 12). Plasma membrane tension involves a multifaceted interplay between the cytoskeleton, membrane-associated proteins, and lipid composition. Notably, bilayer asymmetry contributes to increased stiffening compared to a symmetric membrane, suggesting that lipid remodeling may be a critical factor in biological processes involving exocytosis and endocytosis (13, 14).

In this review, we discuss the role of PLs rearrangement at the plasma membrane during exocytosis and endocytosis. We focus on PLs that primarily serve as structural components of the plasma membrane including PC, PE, and PS. They are contrasted with signaling lipids, which are synthesized in response to a signal (mainly phosphorylated PI derivatives, PA and diacylglycerol (DAG)) and present at lower concentrations at the plasma membrane (reviewed in (15, 16, 17)). These signaling lipids often serve as molecular beacons to recruit and activate key proteins involved in membrane trafficking, although some also exhibit additional structural roles (15, 18, 19). We also examine the role of cholesterol in regulating membrane properties in conjunction with structural PLs. After a brief overview of the mechanisms governing plasma membrane asymmetry, we discuss how the loss of this asymmetry characterized by the externalization of inner-leaflet lipids and altered lipid distribution affects the coupling between exocytosis and endocytosis. We place particular focus on neurosecretory cells, specialized cell types capable of synthesizing, storing, and releasing hormones and neurotransmitters critical for regulating diverse physiological processes.

## Control of the plasma membrane asymmetry: An overview

Membrane asymmetry in eukaryotic cells is not an intrinsic property of the lipids but rather a dynamic state acquired and maintained through the orchestrated activity of various lipid transporters and enzymes that selectively distribute lipid species across the bilayer. In animal cells, most enzymes responsible for lipid biosynthesis reside on the cytoplasmic leaflet of the endoplasmic reticulum (ER) membrane (20). From there, structural PLs are delivered to other compartments through both vesicular and non-vesicular transport pathways (21, 22, 23). Although the exact distribution of structural PLs across the ER membrane remains debated, it is well established that membrane asymmetry primarily arises in the Golgi apparatus and is maintained throughout the secretory and endocytic pathways, where flippases, floppases, and scramblases play crucial roles (24, 25, 26, 27, 28). Notably, a recent study integrating cellular, biochemical, quantitative lipidomics, and computational approaches strongly supports the notion that cytoplasmic leaflets of human erythrocyte membranes have more than 50% overabundance of PLs compared to exoplasmic leaflets (29), reinforcing previous observations that have been overlooked due to perceived biases (30, 31, 32, 33, 34). This imbalance is enabled by highly asymmetric distributions of cholesterol, which can rapidly redistribute to buffer leaflet stresses (29). In addition, PL unsaturation is markedly asymmetric at the plasma membrane, with the inner leaflet containing approximately twice as many unsaturated PL as the outer leaflet (5). Hence, at steady state, the outer leaflet is enriched in saturated PL and cholesterol, making it more tightly packed and less fluid than the inner leaflet. Strikingly, this lipid asymmetry of the plasma membrane is mirrored in the asymmetric structures of transmembrane protein domains, where the transmembrane segments have a larger volume in the inner leaflet than in the outer leaflet (5). These asymmetries are conserved throughout eukaryotes, suggesting they represent fundamental cellular design principles.

Early studies on erythrocytes demonstrated that spin-labeled PE and PS are actively transported to the inner leaflet of the plasma membrane in an ATP-dependent manner *via* P4-type ATPase (“flippase”) (35, 36). In contrast, PC from the inner leaflet is actively translocated outward by ABC transporters (“floppases”) (37, 38). In humans, the P4-ATPases family consists of 14 proteins, most of which are composed of a catalytic α-subunit and a non-catalytic β-subunit of the Cdc50 family of integral membrane proteins (39). ABC transporters form a large superfamily, divided into seven subfamilies (ABCA through ABCG) based on their sequence and structural homology. Although most ABC transporters export a wide range of substrates including PLs and cholesterol from the cytoplasm, some are localized to specialized endomembranes like peroxisomes, lysosomes, or mitochondria (40).

Unlike P4-ATPases and ABC transporters, scramblases mediate non-selective lipid redistribution across the bilayer in an ATP-independent but Ca^2+^-dependent manner, leading to the loss of plasma membrane asymmetry (41, 42, 43, 44). In contrast to flippases and floppases, proteins with scramblase activities are structurally diverse. The first protein supposedly exhibiting scramblase activity, Phospholipid Scramblase-1 (PLSCR1), was purified from erythrocytes and classified as a type II transmembrane protein of 37 kDa capable of scrambling PS, PE, and PC in a Ca^2+^-dependent manner when added to purified liposomes (45, 46). However, the function of PLSCR1 as a scramblase has been questioned, as discrepancies exist between PLSCR1 expression levels and the extent of PS exposure observed in various cell types (47, 48, 49) or model organisms (50, 51). Additionally, it remains unclear how a single-pass transmembrane protein could effectively convey PLs across the membrane (see below).

The search for additional scramblases led to the identification of TMEM16F (Anoctamin6), a Ca^2+^-dependent scramblase responsible for PS egress in erythrocytes and mutated in patients with Scott syndrome, a rare congenital bleeding disorder due to defective PS externalization (42). Another scramblase, XKR-8, triggers PS egress in response to apoptotic stimuli (44). Structurally, both TMEM16 and XKR family members share a hydrophilic groove formed by α-helices to facilitate PLs transport (52, 53, 54). XKR-8 also contains two helices that penetrate halfway into the membrane and a C-terminal tail region, which needs to be cleaved by caspases to activate lipid scrambling during apoptosis (54, 55). More recently, a novel family of potential scramblases has been identified. Indeed, the integral membrane protein insertases, responsible for inserting and correctly folding newly synthesized proteins into the ER membrane, have been found to contain a hydrophilic groove capable of scrambling lipids *in vitro* while simultaneously facilitating the insertion of polypeptide chains into the ER membrane (56).

Although regularly questioned (57, 58), the current model of lipid transport by flippase, floppase, and scramblases takes into account the constraint linked to the amphipathic nature of PLs. In this model, the polar head group interacts with the hydrophilic groove of the protein, while the acyl chains remain embedded within the membrane, analogous to swiping a credit card through a card reader (59). Recently, a new mode of PLs translocation has been proposed for the Voltage-Dependent Anion Channel (VDAC). VDAC proteins are β-barrel proteins that form a pore allowing ATP and other metabolites to cross the outer membrane of mitochondria. Intriguingly, VDAC1 and VDAC2 can form homodimers able to scramble PLs. It was hypothesized that VDAC dimers facilitate PLs flipping by inducing membrane thinning and creating a hydrophilic groove at the dimer interface rather than within the β-barrel itself (60).

The development of genetically encoded fluorescent lipid-binding proteins has allowed the visualization and tracking of many specific lipid species in living cells, providing compelling evidence for the heterogeneous and asymmetric lipid distribution in cell membranes (15, 61, 62). Because PS is normally restricted to the inner leaflet, its exposure on the outer leaflet of the plasma membrane serves as a hallmark of lipid asymmetry collapse, which can be readily and unambiguously detected by high-affinity PS probes.

The loss of plasma membrane asymmetry has been observed in many biological processes, including apoptosis, blood coagulation, muscle cell differentiation or synaptic pruning for example (28, 43, 49) (Table 1). In these contexts, PS exposure is typically massive and sustained, serving as a recognition signal for receptors or other proteins to trigger cellular responses. However, the loss of plasma membrane asymmetry can also be transient and localized, as observed in secretory cells (18, 63, 64, 65). In these cells, membrane asymmetry must be rapidly restored to maintain plasma membrane homeostasis and enable subsequent rounds of exocytosis.

**Table 1:** Characteristics of PS externalization in diverse biological processes

Biological process	PS exposure duration	PS quantity	Process Reversibility	References
Platelet activation and coagulation	Short	Limited	Reversible	(192)
Myoblast fusion	Short	Limited	Reversible	(193)
T cell activation	Short	Limited	Reversible	(194)
Cell-cell fusion in trophoblasts	Short	Limited	Reversible	(195)
Microparticle (vesicle) shedding	Short	Limited	Reversible	(196)
Entosis (cell invasion)	Short	Limited	Reversible	(197)
Regulated exocytosis	Short	Limited	Reversible	(64, 65)
Apoptosis	Long	Massive	Irreversible	(198)
Erythrocyte clearance	Long	Massive	Irreversible	(199)
Macrophage efferocytosis	Long	Massive	Irreversible	(199)
Sperm capacitation and acrosome reaction	Short	Massive	Irreversible	(200)
Neuronal pruning	Long	Massive	Irreversible	(201)
Tumor metastasis	Long	Massive	Irreversible	(202)
Bone mineralization	Long	Massive	Irreversible	(43)

This table summarizes various biological processes associated with phosphatidylserine (PS) externalization, taking into account the relative duration of PS exposure on the outer leaflet (short or long), the relative amount of PS exposed (limited or massive), and whether the plasma membrane returns to its initial state (reversible or not).

## Is PLSCR1 a scramblase?

While the activities of flippases and floppases maintain lipid asymmetry, scramblases uniquely disrupt this balance. Among scramblases, PLSCR1 has drawn significant attention and debate regarding its direct role in phospholipid redistribution, leading to a critical re-evaluation of its function and significance.

**Figure 1:**
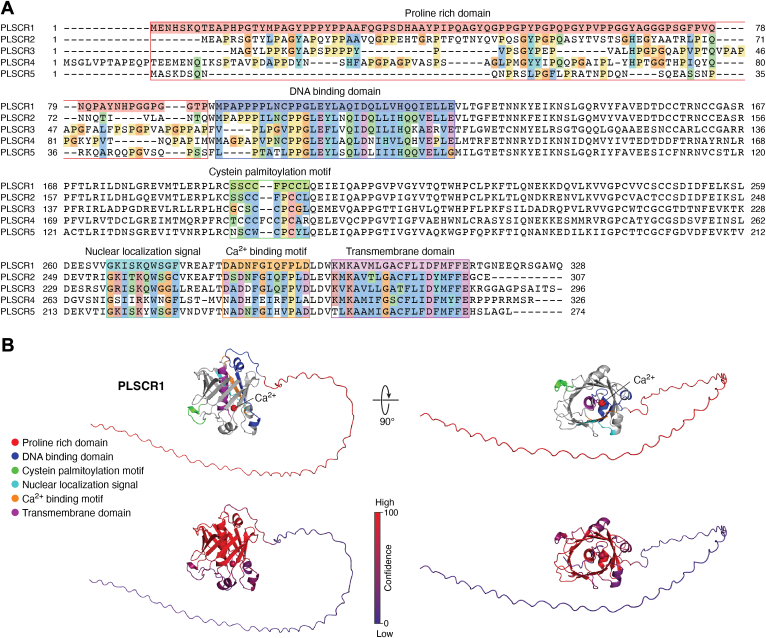
**Functional domains in PLSCR1**. *A*, alignment of mouse phospholipid scramblases (Q9JJ00, PLSCR1; Q9DCW2, PLSCR2; Q9JIZ9, PLSCR3; P58196, PLSCR4; J3QM92, PLSCR5). Functional domains identified based on the PLSCR1 sequence are highlighted: the proline rich domain (*red*, residues 1–93); the DNA binding domain (*blue*, residues 95–127); the cysteine palmitoylation motif (*green*, residues 191–199); the nuclear localization signal (*cyan*, residues 266–275); the Ca^2+^ binding motif (*orange*, residues 282–293), and the transmembrane domain (*purple*, residues 297–315). The alignment was generated using Clustal Omega webserver, and domain annotation was carried out with Jalview. Amino acid conservation is indicated with Clustal color coding, where residues are colored based on their physicochemical properties: hydrophobic (*blue*), polar (*green*), positive charge (*red*), negative charge (*magenta*), glycine (*orange*), proline (*yellow*), aromatic (*cyan*) and cysteine residues (pink). Unconserved residues are white. *B*, structural prediction of PLSCR1 obtained using the Alphafold server. Functional domains are highlighted using the same color scheme as in *panel* A. Structures were visualized and colored in PyMol. The predicted structure displays a β-barrel core with an extended, unstructured prolin-rich region. Notably, the putative transmembrane domain is predicted to be embedded within the β-barrel. The palmitoylation motif protrudes from the surface, consistent with its proposed role in membrane anchoring. Calcium ion is shown as a *red ball*. The right top panel shows the structure rotated by 90° relative to the top *left panel*. Bottom panel corresponds to a per-atom confidence estimate on a 0 to 100 scale (*blue* to *red*) where a higher value (*red*) indicates higher confidence.

Based on sequence similarity, PLSCR proteins (PLSCR1 to PLSCR5; (41, 66)) have been classified as type II single-pass transmembrane proteins with their N-terminal domain facing the cytoplasm (Fig. 1). Due to the lack of reliable antibodies, information on the subcellular distribution of endogenous PLSCRs remains limited. However, with the exception of PLSCR2, which is localized to the nucleus, overexpressed PLSCRs are mostly found at the plasma membrane and in endomembranes (64, 67, 68). This distribution is likely due to their conserved domain organization. PLSCR proteins possess a cysteine-rich region that undergoes palmitoylation, a modification crucial for membrane targeting as well as localization in membrane rafts, where, for instance, PLSCR1 can associate with the EGFR complex (67, 68). The isolated PLSCR1 transmembrane domain can insert into liposomes membranes and displays affinity for cholesterol (69, 70, 71). In contrast, overexpressed PLSCR1 lacking its transmembrane domain is mostly cytoplasmic (70, 72, 73), indicating that the C-terminal sequence and palmitoylation anchor PLSCR1 within cholesterol-enriched domains of the plasma membrane. When palmitoylation is inhibited, PLSCR1 relocalizes to the nucleus *via* a non-classical nuclear localization signal (74). Palmitoylation is thus believed to play a critical role in stabilizing PLSCR1 at the plasma membrane and targeting it to lipid rafts. PLSCR1 also possesses a DNA-binding domain capable of enhancing the transcription and expression of IP3 receptors (68, 75). Additionally, PLSCR proteins are Ca^2+^-sensitive, containing a conserved EF-hand-like calcium-binding domain, essential for their function (76). A less well-conserved proline-rich domain (PRD) is also present in PLSCR1. Notably, although PLSCR2 has no scrambling activity *in vitro*, it was shown to mix lipids when its PRD is swapped with that of PLSCR1 (PRD_PLSCR1_-PLSCR2) (77). In addition, the PRD of PLSCR1 drives PLSCR1 or PRD_PLSCR1_-PLSCR2 oligomerization in the presence of Ca^2+^ *in vitro* (77), suggesting that Ca^2+^- dependent PS egress by PLSCR1 may require oligomerization (46).

The discovery of TMEM16F and XKR-8 as key scramblases responsible for PS egress during blood coagulation and apoptosis, respectively (42, 44), has raised questions regarding the lipid-scrambling function of PLSCR1 (78, 79). This reevaluation primarily emerged from studies using animals lacking PLSCR1 (PLSCR1 KO) or its homologs. For instance, PLSCR1 KO mice display no homeostatic defects and show normal PS exposure upon activation of erythrocytes or platelets (47). Similarly, PS-mediated phagocytic clearance or apoptosis was not compromised in *Drosophila* or *Caenorhabditis elegans*, respectively, when PLSCR1 homologs were deleted (50, 51). These findings suggest that other scramblases may compensate for the absence of PLSCR1. The externalization of structural PLs at the plasma membrane by scramblases is also critical for viral infection (80). While TMEM16F activity is required for SARS-CoV-2-driven syncytia formation (81), PLSCR1 in contrast appears to act as a potent cell-intrinsic antiviral factor, acting by preventing virus-endosome fusion and the subsequent release of viral RNA into the host cell cytosol (82). When PS exposure was assayed, PLSCR1 KO cells treated with calcium ionophore (ionomycin) showed no defects compared with wild-type control, whereas TMEM16F KO cells exhibited a profound defect. Interestingly, overexpressing PLSCR1 in TMEM16F KO cells restored PS exposure, albeit less efficiently. However, mutating the Ca^2+^-binding site or its palmitoylation motif prevented PLSCR1 from restoring PS exposure confirming the importance of Ca^2+^ binding and membrane targeting in its function (82).

Despite the ongoing debate about its direct scramblase activity, several studies indicate that PLSCR1 is required for PS exposure in specific biological processes, particularly in membrane trafficking. For example, using adrenal chromaffin cells and cerebellar neurons from PLSCR1 KO mice, we have shown that PS egress induced by cell stimulation requires PLSCR1 (64, 65). Similarly, mast cell degranulation by antigen-mediated aggregation of the FcεRI triggers PLSCR1-dependent PS egress (83, 84, 85). In the context of viral infection, herpes simplex virus has been shown to activate PLSCR1 and translocation of PS towards the external leaflet of the plasma membrane (86). Collectively, these findings suggest that PLSCR1 contributes, at least indirectly, to plasma membrane PLs scrambling across various cell types and physological processes. Importantly, these findings also highlight that scramblases may function redundantly in some contexts, whereas in others, they may not fully compensate for each other.

Interestingly, structural predictions by AlphaFold2 indicate that PLSCR1 adopts a 12-stranded membrane *β-*barrel in which the C-terminal hydrophobic helix is buried (82) (Fig. 1*B*), a structure reminiscent of VDAC proteins (87). However, the *β*-barrel of PLSCR1 is shorter than that of VDAC1 (25 Å compared to 40 Å), making it unlikely to span the full membrane bilayer unless the plasma membrane’s thickness is drastically reduced. Given its multi-modular nature, PLSCR1 may also regulate other lipid transporter activities, yet to be identified, or function as a signaling scaffold *via* interactions with diverse protein partners (88).

Although PLSCR1’s intrinsic scramblase activity remains unresolved and warrants further investigation, accumulating evidence supports a role for PLSCR1 in modulating plasma membrane lipid topology during specific biological processes, especially in neurosecretory cells, a specialized cellular model frequently used to dissect mechanism of regulated exocytosis and compensatory endocytosis.

## Coupling between exocytosis and endocytosis: A key role for structural PLs

**Figure 2:**
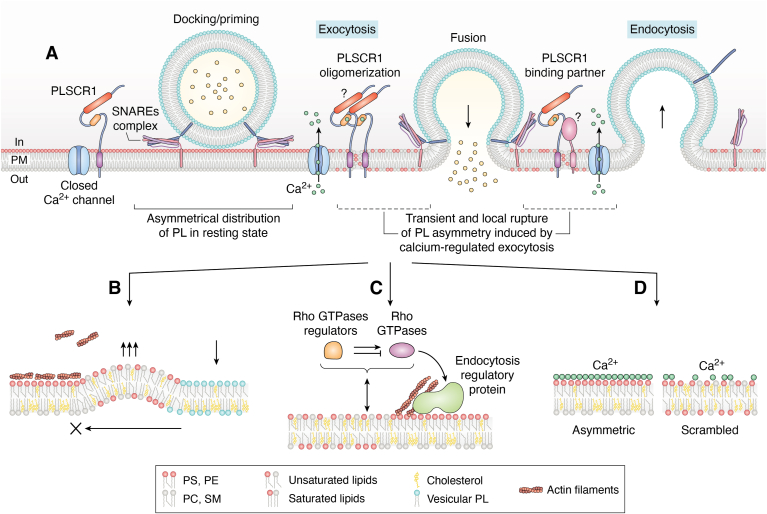
**Phospholipid scrambling and its functional consequences during the exocytosis–endocytosis cycle of secretory vesicles**. *A*, in the resting state, the plasma membrane (PM) exhibits an asymmetric phospholipid distribution of PLs. Ca^2+^ channels are closed, PLSCR1 is inactive, and few secretory vesicles are docked. Upon stimulation, localized calcium influx at fusion sites transiently activates PLSCR1. PLSCR1 may require homo-oligomerization or interact with an unknown partner to transiently scramble PL around exocytic sites. After membrane fusion and cargo release, vesicle components, including vesicular SNAREs, must be retrieved by compensatory endocytosis to replenish the pool of fusion competent vesicles. *B*, At synapses, phospholipid scrambling disrupts lipid asymmetry, reducing membrane stiffness. As the vesicle flattens into the PM, lateral compression preferentially deforms the scrambled membrane. Actin structures limit tension propagation, thereby enabling membrane bending and the initiation of endocytic pit. *C*, Scrambling may also modulate the recruitment of proteins, including Rho GTPase regulators, which in turn influence local Rho GTPase activity and dynamics. This leads to remodeling of the actin cytoskeleton, promoting and activating the endocytic machinery. *D*, the transient loss of asymmetry may also interfere with calcium–lipid interactions, particularly with negatively charged lipids such as PS. This could facilitate the lateral diffusion of lipids and membrane-associated proteins, helping to clear the fusion site and optimize the spatial organization of endocytic effectors.

Exocytosis and endocytosis are tightly coupled processes (Fig. 2*A*) that are essential for numerous cellular functions, including neurotransmission, phagocytosis, immune response, and cell migration. This coupling was first observed 50 years ago in electron microscopy studies on frog neuromuscular junctions, where membrane addition during exocytosis was balanced by compensatory endocytosis (89, 90). Subsequent experiments demonstrated that blocking synaptic vesicle exocytosis in stimulated neurons prevented endocytosis, highlighting the dependency of endocytosis on exocytosis (91). Interestingly, even a brief hyperosmotic shock, which induces calcium- and Synaptotagmin-1-independent exocytosis, is sufficient to trigger compensatory endocytosis, suggesting that vesicle membrane insertion into the plasma membrane itself serves as a direct signal for endocytosis (92).

Depending on the cell type and physiological requirements, the coupling between exocytosis and endocytosis is influenced by multiple biochemical and biophysical constraints, including changes in membrane tension, the diffusion properties of lipids and proteins, and feedback mechanisms that coordinate exocytosis and endocytosis in space and time (11). Despite the crucial role of structural PLs in determining the biophysical properties of membranes, functional data beyond those obtained from simplified *in vitro* systems remain scarce due to the technical challenges of studying lipid behavior in living cells. Consequently, most of our understanding on this matter was actually drawn from *in vitro* studies using vesicles with uniform lipid composition and/or distribution that fail to fully replicate the complexity of cell plasma membrane, where membrane tension is heterogeneous, primarily due to the underlying actin cortex (8, 11). Although the roles of signaling lipids and cholesterol are better documented (18, 93, 94), the specific contributions of other lipids remain less understood. Despite these limitations, experimental evidence strongly suggests that structural PLs contribute to vesicle fusion regulation by influencing fusion pore formation and expansion (95, 96, 97), and in endocytosis by modulating regulatory proteins and membrane tension (8, 98, 99, 100, 101).

### How structural PLs remodeling may impact exocytosis?

According to current models, the core machinery driving membrane fusion is composed of the soluble N-ethylmaleimide-sensitive factor attachment protein receptor (SNARE) complexes localized on opposing membranes. For synaptic vesicles and chromaffin secretory granules, the complex consists of one vesicle-associated v-SNARE, VAMP2, and plasma membrane associated t-SNAREs Syntaxin-1 (one helix) and SNAP-25 (two helices). The helices zipper into a parallel four-helix bundle, whose progressive assembly provides the free energy required to bring the membranes into a close apposition and initiate their merging (102). This process involves the formation of a stalk between interacting membranes, which subsequently elongates laterally and opens into a fusion pore. Live cell imaging of secretory granule fusion with the plasma membrane in chromaffin cells revealed that the fusion pore can adopt a range of metastable sizes, leading to variable rates of cargo release and vesicle retrieval (103). Depending on the elastic energy of the membrane, the fusion pore flickers and may either close or expand as the membranes fully fuse, leading to partial or full release of the vesicle's content (104). Subtle modulation of membrane tension and rigidity is required to either stabilize the stalk structure, maintain the hemi-fusion state, or to promote fusion pore expansion, leading to vesicle release contents (105).

**Figure 3:**
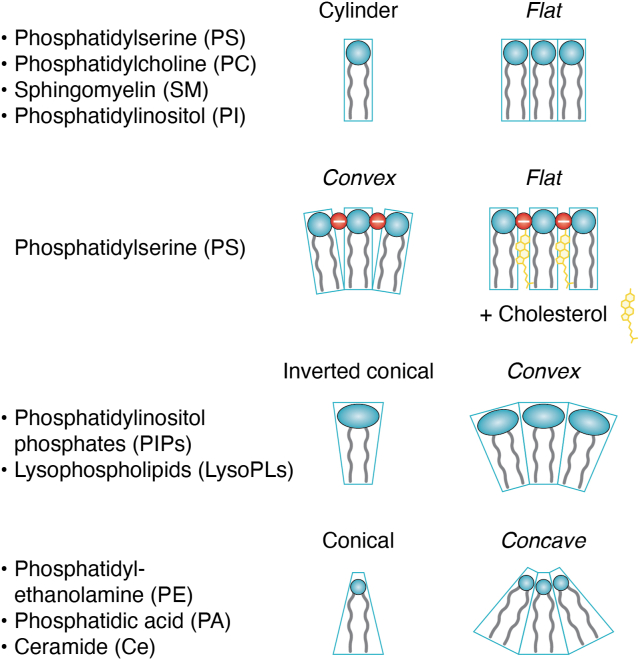
**Scheme of differently-shaped lipids and their associated membrane curvature impact**. Because their headgroup size is similar to that of their hydrophobic tails, lipids such as PS, PC, and SM exhibit roughly cylindrical shape and tend to form flat membranes. However, the repulsive forces of the negatively charged PS headgroup can induce membrane bending. This curvature can be buffered by cholesterol intercalation, which reduces the local charge density. In contrast, PI and lysophospholipids (LysoPLs) have larger headgroups relative to their tails, giving them an inverted cone shape that favors positive (convex) membrane curvature. Conversely, PA, PE, and ceramide (Ce) possess small headgroups, resulting in a conical shape that promotes negative (concave) membrane curvature.

The intrinsic charge and shapes of lipids, determined by their acyl chain compositions and headgroup structure, as well as their location within the bilayers, strongly influence membrane properties (106) (Fig. 3). One of the particularities of the plasma membrane is its rigidity arising from an enrichment in saturated and mono-unsaturated PL and from lipid asymmetry (107, 108, 109) When lipid flip-flop occurs, it generates a symmetric tensionless membrane lowering the energy required for vesicle to fuse with the plasma membrane (110, 111). For example, modifying the distribution of PE in an *in vitro* system with purified secretory granules and planar-supported bilayers (containing SNAREs Syntaxin-1a and SNAP-25) showed that symmetric PE distribution shortens fusion pore lifetime (96). This mechanism may explain why lipid redistribution toward the extracellular leaflet, observed in response to cell stimulation, is required for mast cell degranulation (83, 84).

During regulated exocytosis, SNARE complex assembly is tightly regulated by multiple factors, including synaptotagmins, which act as Ca^2+^-sensors to trigger rapid exocytosis (112). Synaptotagmins bind to PS and, during vesicle fusion, induce lipids demixing, thereby modifying local charge distribution and facilitating fusion (113, 114). In contrast, limiting lipid demixing by increasing membrane tension inhibits fusion by stabilizing the hemifusion stage *in vitro* (115). The relative abundance of structural PLs also influences exocytosis rates. In PC12 cells, excess of PS levels facilitates the fusion pore formation but limits its subsequent expansion (116). Lipid packing, which increases in curved membrane (107), creates a mechanical constraint, which limits the fusion pore expansion. Interestingly, coarse-grained Martini simulations of flat, lipid asymmetric membranes have demonstrated that a vesicle bud can form through the application of lateral pressure. The bud remained stable until lipid-number equilibrium was disrupted by the addition of the TMEM16 F scramblase, resulting in bud shrinkage and bilayer flattening (117). This process resembles the omega-shrink fusion mode (98, 101). Surprisingly, however, abolition of PLSCR1-dependent PS redistribution has no significant effect on secretory granule and synaptic vesicle exocytosis in chromaffin cells and cerebellum granular neurons, respectively (64, 65), suggesting that exocytic machinery-dependent lipid remodeling is sufficient for efficient vesicle fusion in those contexts.

Additional factors influencing exocytosis include the distribution of cholesterol, which is highly enriched in the plasma membrane, where it shapes membrane architecture and regulates cell signaling. Cholesterol induces membrane thickening and lipid condensation, leading to the formation of lipid rafts, dynamic nanoscale regions enriched in cholesterol and sphingolipids (118, 119, 120). Importantly, cholesterol preferentially partitions into negatively curved regions, alleviating membrane stress and reducing bending rigidity (111). However, its biophysical effects depend on lipid saturation: it significantly stiffens saturated membranes while exerting a more moderate effect in mono- or di-unsaturated lipid environments (111). This suggests that the interplay between cholesterol-induced mechanical properties and lipid composition dynamically governs vesicle state transitions, ultimately driving fusion. These properties also influence SNARE protein function. Syntaxins and SNAP-25 form clusters within cholesterol-rich domains, and their activity is tightly linked to local cholesterol availability. Indeed, cholesterol depletion in neurons inhibits calcium-triggered exocytosis while paradoxically promoting spontaneous vesicle release, highlighting its role in balancing exocytotic modes (121, 122, 123). Consistently, single-vesicle fusion assays demonstrate that cholesterol lowers the energy barrier for fusion pore formation, favoring full fusion over hemi-fusion at physiological concentrations (124, 125). Structural studies by NMR further reveal that the extravesicular domain of VAMP2 adopts distinct conformations depending on the lipid environment. Cholesterol-rich domains enhance SNARE complex assembly, whereas acidic lipid domains (containing PS, PI, phosphatidylglycerol and PA) inhibit SNARE activity by promoting VAMP2 extravesicular domain binding to synaptic vesicle membrane and reducing its engagement in the SNARE complex (126). Adding to the complexity of cholesterol’s role into membrane fusion, we recently showed that cholesterol regulates the ability of proteins to bind to PA (127), which is also known to bind Syntaxin1 (128). These findings underscore cholesterol’s ability to regulate exocytosis not only through membrane remodeling but also by modulating SNARE protein conformation and activity.

Beyond cholesterol, the surrounding lipid environment further fine-tunes the regulation of exocytosis. Electrostatic interactions with negatively charged lipids such as PA, PS, and PI(4,5)P2, influence the clustering of Syntaxins and the activity of Synaptotagmins, adding another layer of regulation (128, 129, 130). Among these lipids, PS plays a critical role by contributing to cholesterol retention within the cytosolic leaflet of the plasma membrane, suggesting an interdependent relationship between these lipids (131). Importantly, this relationship appears sensitive to the degree of lipid unsaturation. Indeed, PL unsaturation has been shown to regulate exocytosis by modulating membrane biophysical properties. Monounsaturated PS, for example, induces phase separation in cholesterol-containing bilayers and protects cholesterol from oxidation (131). This biophysical effect extends to SNARE-mediated fusion, where unsaturated acyl chains can bypass the need for Ca^2+^ in Syntaxin1-driven vesicle fusion, likely by disordering membrane lipids to promote SNARE complex rearrangement (132). Supporting this, studies in INS1 cells demonstrate that increasing monounsaturated lipid content enhances the probability of secretory granule fusion (133). Beyond PS, we recently uncovered a differential role for PA based on its degree of unsaturation. PA, synthesized by PLD1 during the exocytosis of secretory granule (134), shows distinct effects depending on its acyl chain composition: mono and di-unsaturated PA species regulate granule docking, while poly-unsaturated PA species modulate fusion pore dynamics (135). Whether PA directly influences cholesterol distribution remains unknown. Nevertheless, as conical shaped lipids (136), PA has been proposed to alter membrane biophysical properties in ways that promote vesicle fusion (137).

Together, these findings highlight the intricate interplay between cholesterol, PL composition, and membrane protein distribution in orchestrating exocytosis. By dynamically tuning membrane properties, these interactions ensure efficient vesicle fusion, which is essential for regulated secretion in various cellular contexts.

### How structural PLs remodeling may impact endocytosis?

Endocytosis is a highly regulated process enabling cells to internalize extracellular material and recycle membrane components. It begins with membrane invagination, orchestrated through the action of specialized proteins and lipids (138). PI(4,5)P2-enriched regions serve as recruitment platforms for key components of the endocytic machinery, while lipid distribution and membrane curvature influence both membrane deformation and vesicle formation. Müller *et al*. demonstrated that addition of PS to ATP-loaded erythrocyte ghosts stimulates endocytic vesiculation, whereas PC, which is not translocated by P4-ATPase flippases of erythrocytes, inhibits this process (139). Further studies revealed that translocating PS or PE from the outer to the inner leaflet of the plasma membrane accelerates both bulk and clathrin-dependent endocytosis (140). Interestingly, a linear correlation was observed between extracellular PS or PE concentrations and endocytic rates, whereas lysophosphatidylserine (LysoPS), which possess a larger polar head showed no effect or even, slowed endocytosis (140, 141). The positive impact of flipped PS and PE on endocytosis, despite their distinct structural and charge properties, suggests that increased lipid concentration in the inner leaflet generates mechanical stress that drives the formation of curved nanodomains. In the case of PS, its negative charge and cylindrical shape favor electrostatic repulsion between densely packed headgroups, promoting negative curvature and the recruitment of curvature-sensitive proteins such as endophilin (142) (Fig. 3). Cholesterol can counteract this effect by stabilizing PS nanodomains and maintaining membrane spacing, but an excess of PS disrupts these interactions, thereby facilitating curvature. In contrast, PE, due to its neutral charge and conical geometry, directly promotes membrane curvature, as shown by its ability to accelerate clathrin-coated bud formation in protein-free liposomes (143) (Fig. 3). In contrast, LysoPS is inducing positive curvature that counteracts plasma membrane invagination required for endocytosis (141).

The role of structural PLs redistribution in endocytosis has also been highlighted by experiments modulating P4-ATPases (flippases), several of which are listed in Table 2 along with their reported functions in vesicular trafficking across organisms. For instance, overexpression of the PC flippase ATP10A results in excessive PC flipping to the inner leaflet, promoting membrane tubulations and increasing β1-integrin endocytosis (144). This indicates that some flippases can dynamically regulate the lipid composition and asymmetry of the plasma membrane, not to necessarily equalize PLs distribution, but rather to modulate membrane properties and functions. In *C*. *elegans*, loss of the P4-ATPase TAT-1 or its chaperone CHAT-1 disrupted PS asymmetry at the plasma membrane and endosomes, leading to defective endocytosis and impaired formation of membrane tubules for sorting (145, 146). In budding yeast, loss of Dnf1p and Dnf2p ATPases induced excess of PE on the cell surface and defective endocytosis (147). Similarly, in mammals, depletion of ATP8A1, (a flippase localized to recycling endosomes), caused dissociation of EHD1 (Eps15 Homology Domain-containing protein 1) and the formation of abnormal, fission-resistant endosomal tubules. Importantly, these defects can be rescued by expression of the ATP8A2 paralog, but not by a flippase-dead mutants of ATP8A2 (148). Furthermore, mutations altering the function of ATP8A2 or ATP9A leading to endocytosis and/or endosomal recycling defects have been associated with neurological disorders (149, 150).

**Table 2:** Flippases (P4-ATPase), floppases (ABC transporters) and scramblases involved in membrane trafficking

Organism	Protein	Type	Function	Substrate	References	Localization
Yeast	Drs2	P4-ATPase	Late Golgi to endosome trafficking	PS > PE	(39)	Trans-Golgi network
Yeast	Dnf1, Dnf2	P4-ATPase	Endocytic vesicle formation	PC, PE	(59, 147)	Plama membrane
Yeast	Dnf3	P4-ATPase	endosomal trafficking	PE > PS	(203)	Late Golgi, endosome
Yeast	Neo1p	P4-ATPase	Membrane trafficking		(204)	Golgi, Endosomes
*Caenorhabditis elegans*	TAT-1	P4-ATPase	Endocytic cargo sorting and recycling		(145, 146)	ER, plasma membrane
*Arabidopsis thaliana*	ALA1	P4-ATPase	Cold stress response, putative trafficking role	PS > PE	(205)	Plasma membrane
*Arabidopsis thaliana*	ALA2	P4-ATPase	Maintains phospholipid asymmetry	PS > PE	(205)	ER, plasma membrane
*Arabidopsis thaliana*	ALA3	P4-ATPase	Golgi to secretory vesicle formation	PS, PE	(206)	Plasma membrane
Mammalian cell	ATP8A1	P4-ATPase	Endosome fission, vesicle formation, neuronal function	PS > PE	(148, 207)	Golgi, Plasma membrane, endosomes
Mammalian cell	ATP8A2	P4-ATPase	Vesicle trafficking in neurons	PS > PE	(208)	Golgi, recycling endosome
Mammalian cell	ATP8B1	P4-ATPase	Bile acid transport, membrane stability	PC,PS,PI	(209)	Plasma membrane, lysosome
Mammalian cell	ATP8B2	P4-ATPase	Role in neural function, unknown pathology	PC,PI	(210)	Plasma membrane
Mammalian cell	ATP8B3	P4-ATPase	Sperm motility and acrosome reaction	PS, PE	(210)	Plasma membrane
Mammalian cell	ATP8B4	P4-ATPase	Associated with autoimmune diseases	PS, PE	(210)	ER, Golgi
Mammalian cell	ATP9A	P4-ATPase	Membrane recycling, endosomal trafficking	PS	(210)	Endosomes, TGN
Mammalian cell	ATP9B	P4-ATPase	Intracellular trafficking	PS	(210)	TGN
Mammalian cell	ATP10A	P4-ATPase	Potential role in vesicle trafficking and neurological disorders	PS, PE	(210)	Golgi, endosomes, plasma membrane
Mammalian cell	ATP10B	P4-ATPase	Parkinson’s disease	PC, GlcCer	(210)	Late endosomes, Golgi
Mammalian cell	ATP10D	P4-ATPase	Lipid metabolism, obesity-related functions	GlcCer, PE	(210)	Golgi, plasma membrane
Mammalian cell	ATP11A	P4-ATPase	Maintaining plasma membrane asymmetry	PS > PE	(210)	Plasma membrane
Mammalian cell	ATP11B	P4-ATPase	Vesicle trafficking and drug resistance	PS > PE	(210)	Golgi, endosomes, plasma membrane
Mammalian cell	ATP11C	P4-ATPase	B-cell development and apoptosis regulation	PS > PE	(210)	Plasma membrane, B cells
Mammalian cell	PLSCR1	Ca^2+^ activated Scramblase	Phospholipid scrambling; cell signaling, gene regulation and compensatory endocytosis	PS, PE	(76)	Plasma membrane
Mammalian cell	PLSCR2	Ca^2+^ activated Scramblase	Function not well characterized; potential role in testis-specific processes		(77)	Nucleus
Mammalian cell	PLSCR3	Ca^2+^ activated Scramblase	Apoptosis regulation and mitochondrial lipid homeostasis		(211)	Mitochondria
Mammalian cell	PLSCR4	Ca^2+^ activated Scramblase	Poorly characterized; may affect signaling		(212)	Plasma membrane
Mammalian cell	TMEM16F (Anoctamin 6)	Ca^2+^ activated Scramblase	Exposes PS during blood coagulation and apoptosis	PS, PE	(42)	Plasma membrane
Mammalian cell	TMEM16K (Anoctamin 10)	Ca^2+^ activated Scramblase	Endosomal trafficking	PS, PE	(25)	ER-endosome contact sites
Mammalian cell	XKR8	Caspase-activated Scramblase	Exposes PS during apoptosis	PS	(44)	Plasma membrane
Mammalian cell	XKR4	Caspase-activated Scramblase	Function in neural tissues	PS	(213)	Plasma membrane
Mammalian cell	XKR9	Caspase-activated Scramblase	Function in gastrointestinal tissues	PS	(213)	Plasma membrane
Mammalian cell	XK	ATP-activated Scramblase	Linked to chorea-acanthocytosis	PS	(214)	Plasma membrane
Mammalian cell	TMEM63B	Mechano sensitive Scramblase	Responds to membrane tension changes	PS, PE	(215)	Plasma membrane
Mammalian cell	ABCB1 (P-glycoprotein, MDR1)	ABC transporter	Efflux transporter in drug resistance	PC, SM, Cholesterol	(216, 217)	Plasma membrane
Mammalian cell	ABCB4 (MDR3)	ABC transporter	Transports phosphatidylcholine into bile	PC	(59, 217)	Plasma membrane
Mammalian cell	ABCC1 (MRP1)	ABC transporter	Exports glutathione conjugates	PC	(218)	Plasma membrane
Mammalian cell	ABCA1	ABC transporter	Cholesterol efflux	PC, SM, Cholesterol	(219)	Plasma membrane, endosomes
Mammalian cell	ABCA7	ABC transporter	Phagocytosis and lipid efflux	PC, SM	(220)	Plasma membrane, endosomes
Mammalian cell	ABCG1	ABC transporter	Cholesterol transport	PC, SM, Cholesterol	(221)	Intracellular vesicles, plasma membrane

This table lists proteins involved in phospholipid transport and scrambling across different organisms, with reported functions in vesicular trafficking. When known, the transported lipid species are also indicated.

Beyond PLs redistribution, the degree of acyl chain saturation may also play a crucial role in endocytosis. For instance, polyunsaturated fatty acids (PUFAs) enhance clathrin-mediated endocytosis of transferrin in non-neuronal cells as well as both constitutive and compensatory endocytosis in neuronal cells (151). Consistently, studies in *C*.*elegans* reveal that the absence of long chain PUFAs in *fat-3* mutants (a delta-6 fatty desaturase enabling the conversion of shorter chain precursor into long chain PUFA) impairs synaptic endocytosis by reducing Synaptojanin recruitment at release sites (152). Collectively, these findings suggest that both PS dynamics and the membrane lipid composition are integral determinants of endocytic efficiency.

### How structural PLs may coordinate the transition from exocytosis to endocytosis?

The coupling between exocytosis and endocytosis is crucial for cellular processes requiring rapid and repetitive membrane turnover. This is particularly critical in systems with limited membrane supply, such as immune cell signaling, hormone secretion and synaptic transmission. At synapses, where vesicle availability must match neuronal firing frequencies ranging from a few Hz to several hundred Hz, rapid vesicle recycling is vital to maintain neurotransmission fidelity. Studies across various synapses and animal models suggest that conserved regulatory mechanisms govern this process (153). While different modes of exocytosis have been well characterized in neuroendocrine cells, synaptic vesicle fusion modes remain debated (98, 154). However, there is broad consensus regarding the retrieval mechanisms following exocytosis, especially in neurons. Depending on stimulation frequency and intensity, four major types of endocytosis have been proposed, primarily classified based on their reliance on clathrin. These mechanisms have been extensively reviewed (153, 155). Clathrin-mediated endocytosis (CME) is considered relatively slow (10–20 s) and typically occurs under intense supraphysiological stimulation (156). CME plays a crucial role in replenishing the reserve vesicle pool (157) and in completing faster clathrin-independent mechanisms, which may be insufficient to fully retrieve all synaptic vesicle components remaining in the plasma membrane (158, 159). In contrast, clathrin-independent endocytosis mechanisms predominate under moderate stimulation, occurring within hundreds of milliseconds after exocytosis (156, 160, 161). Among these modes, ultrafast endocytosis (UFE), is temperature-dependent and relies on dynamin and actin, which both regulate the assembly of endocytic sites, membrane stiffness and molecule diffusion, key properties essential for successful UFE and efficient compensatory endocytosis (156, 160, 162, 163, 164). Nonetheless, recent findings have raised caution regarding the strict classification of endocytic pathways as clathrin-independent, suggesting that minimal levels of clathrin may still be required (165).

Our studies in excitatory cells, using extracellular PS staining as a proxy for membrane asymmetry, have implicated PS dynamics in secretory granules and synaptic vesicles recycling through compensatory endocytosis (64, 65). Although an increase in intracellular calcium level, the direct trigger of exocytosis, also induced PLSCR1-dependent PS translocation, only endocytosis is impaired in PLSCR1 knock-out cells, while the exocytosis rate remains unchanged (64, 65). The critical role of the transient loss of plasma membrane asymmetry in coupling exocytosis to endocytosis is not restricted to neurosecretory cells, as TMEM16F-mediated PS redistribution similarly promotes T cell receptor endocytosis in immune cells (166, 167).

Although the disruption of PLs asymmetry at the plasma membrane is transient during the exocytosis-endocytosis cycle, the precise timing of this asymmetry loss and its restoration remains unclear. One possible explanation is that this transient disruption may create biophysical boundaries between the plasma membrane and the fused vesicle, thereby limiting vesicular lipids and proteins diffusion into the plasma membrane and preventing their dilution (Fig. 2*B*). Additionally, since endocytosis is impaired when flippase function is disrupted, our observations could also suggest that PS egress itself may act as a signal to initiate endocytosis through the subsequent re-establishment of asymmetry *via* flippases function.

Beyond lipid asymmetry, the mechanical properties of the membrane play a critical role in these processes. Synaptic vesicle membranes are enriched in cholesterol, sphingomyelin, and specific transmembrane proteins, which increase their bending modulus and make them stiffer than the plasma membrane (162). This stiffness resists complete flattening during fusion, creating mechanical energy that deforms membrane. In contrast, being more flexible due to its lower bending modulus, the plasma membrane then deforms under lateral compression generated by multiple vesicles fusions, leading to endocytic pits formation (Fig. 2*B*). The decrease in membrane tension following vesicle fusion accelerates pit formation (168), while actin surrounding the active zone constrains tension propagation, thereby enabling UFE to proceed efficiently. Simulations confirm that UFE is only efficient within a specific range of membrane stiffness (162, 163). Membrane asymmetry significantly contributes to membrane stiffening compared to symmetric membranes. Therefore, the rapid flipping of membrane lipids such as PS or cholesterol may help to reduce membrane tension and promote the deformability required for endocytosis (110, 169). Additionally, high content cholesterol in synaptic vesicles restricts the lateral proteins and lipids diffusion during regulated exocytosis (170), yet, such diffusion is necessary to clear the release sites for the next vesicle fusion (171). The clearance of release sites in the presynaptic active zone represents a major rate-limiting step for sustained synaptic transmission (9). During high frequency stimulation, the accumulation of SNARE components at the plasma membrane may constitute a kinetic bottleneck, preventing the next synaptic vesicles recruitment (172). Therefore, asymmetry loss, which increases membrane fluidity by outward redistribution of loosely packed lipids (PE, PS) to the outer leaflet, may be advantageous for sustaining synaptic activity, as it could facilitate rapid protein diffusion and site clearance. Notably, PLSCR1 expression is higher in the cerebellum and the olfactory bulb, two brain regions known for sustaining high-frequency stimulation (65).

Although scramblases are not specific to PS, its abundance and negative charge make it a strong candidate for modulating membrane. PS electrostatic properties are central to multiple regulatory processes, including protein interactions, calcium-mediated membrane stabilization, and actin dynamics (61).

Given the essential role of actin in compensatory endocytosis at synapses, it is noteworthy that the negative charge of PS influences interactions with the cortical actin cytoskeleton. PS redistribution may weaken membrane-cytoskeleton coupling, thereby enhancing membrane flexibility, protein and lipid diffusion, and vesicle retrieval (173, 174). Furthermore, studies in yeast have demonstrated that the redistribution of PS is crucial for regulating exocytosis and endocytosis during bud formation and polarized growth. Notably, flippase-mediated translocation of PS modulates the spatial activation of Cdc42 (a key Rho GTPase in cell polarity), by influencing its dissociation from the guanine-nucleotide dissociation inhibitors (GDIs). This regulation ensures the rapid cycling of Cdc42, maintaining its precise localization at the plasma membrane to support sustained cell polarity (175). Moreover, PS enrichment within Cdc42 nanodomains stabilizes the GTPase in regions of active exocytosis, facilitating vesicular trafficking while counteracting diffusion-driven dilution (176). The differential distribution of PS at sites of exocytosis and endocytosis establishes distinct microdomains, enabling efficient vesicle fusion and selective membrane retrieval through endocytosis (177). Interestingly, Rho GTPases cycling also coordinates exocytosis and endocytosis in mammalian cells (178). In non-neuronal cells, Cdc42 and its regulators control fast endophilin-mediated endocytosis (FEME), a clathrin-independent endocytosis triggered by activation of specific receptors to fine-tune signaling (179). In neuroendocrine cells, intersectin-1L (a Cdc42 guanine nucleotide exchange factor (GEF)) regulates secretory granule exocytosis (180), whereas the Rho GTPase-activating protein (GAP) OPHN1 couples exocytosis to endocytosis by regulating RhoA activity levels (181). More recently, studies in neurons have shown that Rac1 activation and its effector Arp2/3 reduce the replenishment of the synaptic vesicle pool, whereas the RhoA effector mDia1 facilitates this process. The interplay between RhoA and Rac1 activity dynamically regulates actin polymerization, orchestrating the coupling between exocytosis and endocytosis (182, 183) (Fig. 2*C*). Finally, by interacting with the SNARE complex, Intersectin-1 also enables clearance of presynaptic release sites to sustain high-frequency neurotransmission (172). These mechanisms highlight the intricate relationship between lipid redistribution, Rho GTPase regulation and cytoskeletal dynamics in shaping cellular polarity and trafficking efficiency.

Additionally, as a charged PL, PS modulates lipid diffusion by homogenizing the physical properties of the membrane leaflets and reducing the “glue” effect of intracellular Ca^2+^, whose concentration rises dramatically with repeated stimulation (184) (Fig. 2*D*). Overall, these properties underscore the pivotal role of PS in regulating membrane dynamics and cellular adaptability. However, further studies are needed to determine the precise spatiotemporal regulation of lipid flipping at the synapse. In neuroendocrine cells, we have shown that PLSCR1-dependent PS egress occurs at the periphery of the fusion site where it may regulate endocytosis and help preserving the integrity of the secretory granule protein and membrane for efficient endocytosis (64, 185, 186, 187). The underlying molecular mechanisms, however, remain to be elucidated.

## Concluding remarks

The dynamic interplay between exocytosis and endocytosis is fundamental to cellular homeostasis and secretory functions. Although the roles of protein complexes in these processes are well established, growing evidence highlights the importance of lipid asymmetry remodeling, regulated by scramblases, flippases, and floppases, as a critical switch controlling membrane trafficking efficiency (Table 2). Yet, our current understanding of these lipid-mediated mechanisms remains limited, primarily due to technical challenges in monitoring lateral and interleaflet lipid distribution and dynamics with sufficient spatiotemporal resolution. Many conventional techniques often rely on indirect visualization using lipid probes, which may perturb their native function and dynamics. Furthermore, the precise lipid composition of membranes, particularly the degree of lipid saturation, remains difficult to access despite its potential functional relevance (133, 135).

Emerging technologies are opening promising avenues to overcome these limitations. Next-generation fluorescent synthetic PLs (188) combined with super-resolution imaging and fluorescence correlation spectroscopy (FCS), now enable real-time tracking of lipid dynamics in live cells with minimal perturbation. These tools will be instrumental in precisely delineating the contribution of membrane asymmetry and its re-establishment throughout the exo-endocytosis cycle. In parallel, the use of caged lipids allows for temporally controlled lipid release, offering new insights into lipid behavior in physiological contexts (189). Complementary to these approaches, mass spectrometry imaging, although still awaiting subcellular resolution, promises to provide critical information on lipid species composition (190, 191), further refining our understanding of lipid involvement in membrane trafficking. Together, these technological advances hold great promise for uncovering, with unprecedented precision, the complex roles of lipids in exo-endocytosis and beyond.

## Conflict of interest

The authors declare that they have no conflicts of interest with the contents of this article.
